# Pandemic perspectives from detained youth during COVID-19: Bridging the knowledge gap for future safeguards

**DOI:** 10.1371/journal.pone.0309179

**Published:** 2024-10-09

**Authors:** April McNeill-Johnson, Zuri Hudson, Brittany Moore, Dumebi Okocha, Megha Ramaswamy, Kimberly Randell

**Affiliations:** 1 Division of Emergency Medicine, Children’s Mercy Kansas City, Kansas City, MO, United States of America; 2 University of Missouri-Kansas City School of Medicine, Kansas City, MO, United States of America; 3 University of Kansas School of Medicine, Kansas City, KS, United States of America; 4 Department of Pediatrics, Children’s Mercy Kansas City, Kansas City, MO, United States of America; 5 Department of Health Systems and Population Health, School of Public Health, University of Washington, Seattle, WA, United States of America; Caleb University, NIGERIA

## Abstract

Despite the worsening health disparities among youth in detention during the COVID-19 pandemic, there has been minimal exploration into the pandemic experiences of detained youth and opportunities for pandemic mitigation. This paper analyzes the perspectives of youth in detention on the pandemic, including the effect of the pandemic on their detention experience and their perceptions about COVID-19 vaccination. The study used purposive sampling to recruit 16 participants (aged 14–17 years) from two juvenile detention centers in the urban Midwest. We conducted semi-structured interviews and analyzed verbatim transcripts using a hybrid deductive-inductive approach and thematic analysis. Four themes emerged: 1) personal experience influenced youth perceptions of pandemic severity and risk; 2) distrust and misconceptions contributed to youth vaccine hesitancy or refusal; 3) desired opportunities and parental opinion motivated youth to get the COVID-19 vaccine; and 4) pandemic mitigation strategies negatively impacted youths’ detention center experience. Study findings identify opportunities for detention centers to minimize the negative impacts of pandemic mitigation strategies on youth in detention, expand vaccination knowledge and uptake, and build trust to positively impact the health and wellbeing of detained youth currently and during future pandemics.

## Introduction

The COVID-19 pandemic amplified existing health disparities among youth in detention centers. Health disparities among detained youth are disproportionately higher than among the general population [1]. Youth in detention is defined as arrested youth in short-term confinement while awaiting formal judgment by the court system. Two-thirds of incarcerated youth have physical health needs. Forty-six percent of incarcerated youth have at least one diagnosed medical condition [2] in comparison to 19% of youth without legal involvement [3, 4]. Due to confined detention settings, detained individuals are more likely to contract COVID-19 [5] as evidenced by higher rates of COVID-19 in prisons and detention centers compared to the general United States population [6]. Before the delta variant surge, the COVID-19 incidence rate in prisons was 31,000 cases per 100,000 individuals, significantly higher than the U.S. population’s rate of 9,300 cases per 100,000. Additionally, the COVID-19 mortality rate stood at 200 deaths per 100,000 in prisons, surpassing the U.S. population’s rate of 81 deaths per 100,000 [7]. Since the onset of the pandemic, U.S. prisons and Immigration and Customs Enforcement (ICE) detention centers have reported approximately 663,196 cases and 3,181 deaths due to COVID-19. Among U.S. state and county youth detention facilities, approximately 3,000 COVID-19 cases were reported among detained youth as of November 2023 [7]. Factors inherent to detention settings, such as barriers to social distancing, communal living, hygiene practices, and daily influx of varied personnel and new detainees contribute to these high case numbers.

Although the emergency period of COVID-19 has ended, the residual effects of the pandemic are ongoing. There is a crucial need to identify and incorporate lessons learned into current policy and practice, ensuring better safeguards for future pandemics and large-scale disasters. Although decarceration, the release of people from detention, was the practice preferred by prisoners’ rights and health advocates to reduce the spread of COVID-19 among incarcerated individuals, detainee releases were minimal. Only 3% of the US detention facilities reduced the population by early release [8]. Additionally, variable implementation of mitigation practices in prison and jail systems, including prevention measures (e.g., masking and hygiene practices), testing, vaccination availability, and provision of basic physical and mental health care, contributed to high rates of deaths and morbidity associated with COVID-19. Although many facilities increased phone/video communication with family or legal counsel and access to COVID-19 testing, particularly among juvenile detention centers, only 7% practiced other social distancing measures and only 33% of residents, primarily those with symptoms, were screened for COVID-19 in both adult and juvenile facilities [8].

Although COVID-19 vaccination may mitigate risk for youth in detention, current data suggest lower vaccination rates for this population. Among the six states that published vaccination numbers for youth detention facilities, most reported that less than 50% of these youth were fully vaccinated. Factors contributing to low vaccination rates in the detention and prison populations are general discomfort, fear of side effects, and understanding of the COVID-19 virus [9]. However, studies to date have not explored perspectives of youth in detention on the drivers of low vaccinations rates. Additionally, it is currently unknown how pandemic mitigation measures as implemented in the detention center setting may have impacted youths’ detention center experiences. These critical gaps in our understanding of youth perspectives may result in missed opportunities to mitigate risk among this population during future pandemics.

Despite the exacerbation of health disparities among detained youth amid the COVID-19 pandemic, there remains a significant gap in our understanding of their experiences during the pandemic. This underscores the urgent necessity to explore youth experiences and perspectives, as this may provide insights that can strengthen current policies and establish more robust safeguards for future pandemics. Thus, the objective of this research was to delve into the viewpoints of detained youth regarding the COVID-19 pandemic, including their attitudes toward the COVID-19 vaccine and the pandemic’s impact on their detention experiences. Understanding the lived experiences of youth in detention centers during the COVID-19 pandemic will enable the development of more effective strategies to reduce pandemic-associated health disparities among this population and inform response to future pandemics.

## Methods

This study stemmed from a more extensive study examining the healthcare experiences and self-determined health needs of youth in detention. In early interviews, participants spontaneously discussed their healthcare experiences during the COVID-19 pandemic. We then adapted the interview guide to include specific questions related to the pandemic, including COVID-19 vaccination, and the pandemic’s effects on the health and general experiences of youth in detention. These additional questions aimed to inform healthcare providers and detention facilities about how to better safeguard this population during future pandemics.

Therefore, this article presents findings from interview segments during which youth discussed COVID-19. We used a descriptive phenomenological methodology to explore the lived experience of detained youth during the COVID-19 pandemic. This methodology was selected to gain an in-depth understanding of detained youths’ perspectives of COVID-19, vaccination during the pandemic, and the impact of the pandemic on the detention center experience.

This original study and adapted interview guide were approved by the hospital and detention centers’ institutional review boards. The researcher (AMJ) verbally provided the study’s purpose and objectives to each participant, with an option for the participant to read a written copy. Participants were informed that participation was voluntary, they could choose to opt out or withdraw from the study at any time, and that their decision to participate in the study or not would not affect their adjudication process or detention stay. If a youth declined to participate or withdrew from the study, they were given the option to complete an anonymous demographic REDCap survey to enable us to assess potential differences among non-participants. Informed verbal assent was obtained from study participants. A waiver of parental permission was granted as the study presented no more than minimal risk to participants and could not practicably be conducted without the waiver. Confidentiality was protected through deidentification and secure storage of all information.

### Study participants and recruitment

Typical case purposive sampling was used to select youth participants (N = 16) detained in two urban midwestern youth detention centers between January 2021 and March 2022 as the study aims were unique to youth confined during the pandemic [10]. Participants were English-speaking youth aged 14–17 years. Youth who were wards of the state, had severe cognitive delay, or had acute psychiatric complaints were not eligible.

To recruit participants, designated detention center staff identified eligible youth during intake at the center and provided access to a plain-language informational pamphlet about the study. Staff asked if the youth would be interested in meeting with the researcher to learn more about the study. The researcher (AMJ) met with interested youth on scheduled visit days to provide additional information about the study, answer any questions the youth had, and invite participation. The researcher introduced herself as a physician and researcher. All participants who met the inclusion criteria and agreed to meet with the researcher ultimately decided to participate in the study. Detention center staff did not track the number of youths who declined to meet with the researcher.

Several measures were implemented throughout the study to mitigate coercion and ensure voluntary participation and comfortability. Participants were assured of anonymity and confidentiality regarding their responses, except in cases where disclosures of suicidal ideations or reports of abuse necessitated appropriate interventions and reporting procedures. Additionally, the researcher made efforts to establish rapport with each participant, emphasizing their role as a medical professional with a sincere interest in the participants’ well-being. Interviews took place in an easily accessible, neutral environment. Participants were free to share their thoughts without a staff member directly in the interview space. These efforts aimed to create a supportive and non-coercive environment conducive to open and honest participation.

### Data collection

A single study team member (AMJ) trained in qualitative methods conducted face-to-face interviews using a semi-structured interview guide developed by the study team. Interviews were conducted from January 23, 2021 to March 31, 2022 in a private setting at the detention center, lasted up to sixty minutes, and were audio recorded. Participants completed an anonymous demographic questionnaire after the interview.

### Data analysis

A hybrid deductive-inductive approach and thematic analysis informed by phenomenological methodology was used to examine the participant narratives [9, 10]. We analyzed verbatim interview transcripts (after redacting potentially identifying information) using DeDoose (Version 9.0.17). The senior researcher (AMJ) and another coding team member (ZH) individually coded two initial transcripts, then met to create a codebook through consolidation of codes and resolution of discrepancies. The data analysis was structured around broad conceptual categories derived from the research question and objectives, serving as flexible guides throughout the coding process. Each remaining transcript was independently coded by two coding team members (AMJ, ZH, DO) with new codes added when needed. The coding team met regularly to iteratively refine the codebook, resolve discrepancies through discussion and transcript review, and discuss emerging themes.

Upon completion of coding, a single team member (AMJ) then reviewed and clustered codes, incorporating this into emergent themes to paint a descriptive picture of the participants’ perspectives. These themes and illustrative excerpts were reviewed and then refined by the entire study team [11–13]. Due to the confined setting and the high rate of turnover in detention centers, participants’ feedback on our analysis was not feasible.

## Results

Of the 16 youth who were invited to participate in the study, all agreed to participate and completed the interview and questionnaire. Table 1 provides demographics of these youth. We identified four main themes: 1) personal experiences influenced perceptions of the pandemic severity and risk, 2) distrust and misconceptions contributed to vaccine hesitancy or refusal, 3) desired opportunities and parental opinion motivated youth to get the COVID-19 vaccine, and 4) pandemic mitigation strategies negatively impacted the detention center experience.

**Table 1:** Participant demographics.

Participants (n = 16)	N (%)
Age, mean	15.7
Last grade completed	
7th	2 (12.5)
8th	4 (25.0)
9th	3 (18.7)
10th	3 (18.7)
11th	1 (6.3)
12th	1 (6.3)
N/a	2 (12.5)
Gender	
Female	5 (31.0)
Male	11 (69.0)
Ethnicity	
Hispanic	4 (25.0)
Non-Hispanic	12 (75.0)
Race	
African American	7 (38.0)
Caucasian	3 (25.0)
American Indian	1 (6.5)
Mixed-race	2 (12.0)
Prefer not to answer	2 (12.0)
Unsure/Unknown	1 (6.5)

### Theme 1: Personal experience influenced perceptions of pandemic severity and risk

Detained youths’ perceptions of the legitimacy and gravity of the pandemic were rooted in their personal encounters with COVID-19 and messages they received from family, friends, and media outlets. We identified youth who believed COVID-19 to be a real problem as well as youth who believed the virus was not real or not a serious threat to the public’s health.

### Subtheme 1a: COVID-19 was a significant issue

Many participants thought COVID-19 was a significant issue. These perspectives on the pandemic were shaped by having contracted COVID-19 personally, having loved ones who were impacted by COVID-19, or the physical surroundings youth found themselves in during the pandemic. The participants described their experiences while detained and while in the general population. A youth who was infected with COVID-19 eight months into the pandemic shared concern about contracting the virus again: "I just don’t want to get that. I feel like it’s dangerous, and it affects your immune system. It just affects you in many ways, like your taste, smell, and headaches. I heard your hair starts falling out." The way that young people managed the pandemic and perceived the threat of the virus was influenced by individualized experiences.

Participants also discussed the impact of their family members having the illness. One participant whose mother contracted COVID-19 shared, "It was bad…[S]he had back pain too. She just felt like it was taking her over, and she didn’t even cook no more. She just had to sit down, lay down. She would just sleep all the time. She was just trying to feed herself, give herself tea, eat hot stuff." Another participant shared that he recognized that COVID-19 was real when his brother, who was also at the detention center, contracted the virus but did not exhibit any symptoms and was found incidentally when he was detained: "Man, I’ve heard stories of it not being real, but my brother just had COVID. He came [to the detention center nurse] about something else, and then they told him he had COVID. So, they had to quarantine him and stuff. But he’s good now." Although their family members were impacted to differing degrees by the pandemic, among these participants, family member’s experiences influenced their belief that the COVID-19 virus was real and significant.

As participants recounted personal and familial experiences with the virus, some described changing their personal hygiene practices and taking other precautions to mitigate COVID-19 in response to their contact with other individuals and their environment. One participant explained why she continued to wear a mask while in detention, although detention center policy did not dictate wearing one. She shared, "You don’t even need to wear a mask. But me, I still wear a mask though. Staff come in here [detention center] from outside, so I just still keep my mask on." Despite varying experiences with its effects, participants viewed the pandemic as real and adapted their behavior accordingly.

#### Subtheme 1b: COVID-19 is fictitious or not a serious public health concern

A smaller number of participants thought that the pandemic was fictitious or not a serious public health concern. Many of the participants did not think COVID-19 or the health risks associated with the virus were real, largely due to skepticism, personal experiences with less severe virus symptoms, or misinformation in the media. Participants were skeptical that an unanticipated novel virus could spontaneously appear. As shared by one participant, "How did Corona just up and came out of nowhere? People are saying it came from someone eating a bat. Some people would say, ’Oh, it came from this and that.’ I don’t know how it came [about], so why should I believe something that I don’t have so much information on. I think it’s fake, I think it is man-made." Additionally, some participants thought it was not a serious problem because the people they knew who had contracted COVID-19 did not have severe side effects. A participant with firsthand experience with the virus said, "I had it before, and I didn’t die or nothing. My mama had it. She didn’t get hurt awful, and she [already] got bad health. So, I think it’s just like another flu." Another participant shared, "I just feel like I can’t get it. I been around people that had COVID, and I never got it. I mean, somebody had it here [detention center], and I was in his face, and we was playing basketball together. He coughed, and I ain’t get it. I just feel like I got a good immune system.”

Another participant felt that getting sick with COVID-19 was the fault of the individual: "I don’t think nobody can actually really die from COVID. I don’t know. I just don’t believe it. They got something wrong with their body. That’s how I feel." Media outlets affected another participant’s perception of the pandemic. She believed reports on COVID-19 were exaggerated and reports of pandemic related deaths were made up. She shared how information from a news outlet about death reports impacted her perspective: "Because when COVID was actually happening last year, say, for instance, if somebody got into a car wreck, they would [say], ’Oh, COVID. It was Coronavirus.’ They would even put on their death certificate that he died from Coronavirus." The perceived authenticity or severity of the pandemic among youth was directly impacted by the pandemic related information they accessed and individualized experiences.

### Theme 2: Distrust and misconceptions contributed to vaccine hesitancy or refusal

Most participants said they would not get the COVID-19 vaccine, even among those who felt that COVID-19 was a legitimate concern. They shared several concerns and perceptions that contributed to overall distrust or perceived lack of need for the vaccine.

#### Subtheme 2a: Concern about the speed of vaccine development contributed to distrust

Participants expressed concern about the speed of vaccine development. One participant who preferred to wear a mask to prevent spread of COVID-19 in lieu of vaccination shared, "I don’t really trust the vaccine because they made it too fast. It takes years to make that kind of stuff and they just made it too fast.…I don’t know if it might cause any health problems in the future, so I just don’t want to take it." Another participant with similar concerns shared, "Because usually when they make medicine, it takes like four or five years. This only took couple months…. I just don’t trust it. If they would’ve took their time making it, I would feel more comfortable." Another participant expressing concern about the speed of vaccine development described a lack of understanding of the process and of the vaccine components: "I’ve been hearing it has the COVID in it. To cure COVID is to use it. I don’t want it because I don’t understand that process." COVID-19 vaccine development was authorized for emergency use at the beginning of the pandemic, and even though the public was assured about the safety of the vaccine, youth participants did not have full confidence in its development.

#### Subtheme 2b: Misconceptions about potential vaccine side effects and susceptibility to illness swayed youths’ opinions

Participants shared misconceptions about potential vaccine side effects and susceptibility to illness. One youth who had an uncle detained in an adult prison during the pandemic shared his concern about the side effects of the COVID-19 vaccine: "I just know some people say it make you act crazy…they tried to give my uncle, he incarcerated, so they tried to give him the vaccine so he can have visitors and stuff like that. But he said no because it makes you act crazy." Another participant expressed her unwillingness to get the vaccine by saying, "I think it will mess up your body. I think it’s the same thing as pumping yourself with drugs. I seen a girl that went paralyzed from it." Another participant believed vaccination was unnecessary because being in detention made him less likely to contract the virus: "I would [get the COVID-19 vaccine], but I’m not going to, because I don’t feel like I need it, for real…I haven’t got [COVID-19], so I just don’t figure I’m going to get it. Especially in [the detention center], I don’t feel like I’m going to get COVID from anybody." Another participant thought that their personal mitigation strategies were more likely to shield them from disease than the vaccine: "I feel like I don’t need it. I feel like it gives people COVID…I’ve seen people get vaccinated, and you could still get it. It’s just more of a guarantee that you’re not getting [it bad], I feel I could just be clean and not get it. I always have my hands clean, not touch my face, keep my mask up." Participants stated that most of the information they learned about the COVID-19 vaccine came from friends, family, and social media; this information shaped their perceptions and affected vaccine hesitancy.

### Theme 3: Desired opportunities and parental opinion motivated youth to get the COVID-19 vaccine

Youth cited various reasons they could be persuaded to take the COVID-19 vaccine. Most were related to perceived personal gain, but a few were secondary to parental requirements. Participants assented to vaccination as it facilitated privileges, opportunities, and activities that had been restricted during lockdown. One participant expressed that although neither she nor her family believed in COVID-19, she chose to be vaccinated due to her family’s desire to go on vacation. She said: "Because now, if you want to go on an airplane, you have to show them your vaccine card. And we were all planning to go to Florida. So, we were just like, ’Might as well.’ But if we also get, I’m pretty sure like a cold or something, that vaccine could fight some of it off." A participant discussed how he did not want to get the COVID-19 vaccine, but he was not able to play sports without it: "My mom told me I had to get it to participate in sports."

Participants expressed that parental desire was a major factor in getting vaccinated. A participant who felt his mother forced him to get the vaccine shared, "My mama forced me to get both of them [vaccination series] because when I was going to school, they had came there with a little paper you had to sign, and my mama signed it." Another participant who had been thinking about getting the vaccine wanted parental input before deciding: "I’m 50/50. I feel like my mom will have to approve [getting the vaccine]. I feel like I got to talk to my mom." Parental influence and participation in preferred activities played a significant role in COVID-19 vaccination among young people.

### Theme 4: Pandemic mitigation strategies negatively impacted the detention center experience

Youth described the negative impacts of the pandemic on their detention experiences. Visitation was limited with bans on personal touch, halts were placed on packages from the outside community, and a 10-day quarantine was mandated at detention entry.

#### Subtheme 4a: Limits on visitation and contact while detained during the pandemic

Youth participants described limits on visitation and contact while detained during the pandemic. During the height of the pandemic, visitation was not permitted in the detention centers. A youth with a history of multiple detainments expressed his distress due to decreased interaction with his family while in detention: "The last time I was locked up I couldn’t see my people because of the COVID. You don’t get visitations. Even whenever I was at [the residential facility]. I was there for a while, and they wouldn’t let me [have visitations]–you normally get to go on home passes too. You leave on the weekends to go back to your home. And then none of that because of COVID. It was all over phone calls. And hardly people never answered the phone half the time. So, it was just barely got to talk to my people." When the facility was not in full lockdown, more lenient but still limited visitation policies were implemented. One participant expressed that although he could have family visit, direct physical contact was not allowed. The participant shared, "I don’t really like it because of the mask and stuff…And then you can’t really visit your family and stuff like that. You can still visit them over there, but you just can’t have physical visits and stuff." Another participant expressed, “At visits, can’t hug our mama or nothing. You got to wear a mask every visit. Orientation, you got to wear a mask. [How does that make you feel?] I don’t even like visits no more. Because I can’t even touch my mama.” Limited contact with loved ones during the pandemic contributed to the negative detention center experience.

#### Subtheme 4b: Limitations on delivery of outside items during the pandemic

Detention center policies placed limitations on delivery of outside items during the pandemic. Youth shared that COVID-19 mitigation measures meant they could no longer receive items from outside the detention center. A participant described the negative impact of this limitation on his personal hygiene and reading selection: "Man, they stop having people bring stuff up here. It affects me because my momma tried to buy me some Colgate toothpaste. But they said no because of COVID." Another participant who expressed reliance on faith during confinement shared, "My grandma tried to bring a bible up here for me and they said no." The increased restrictions on outside deliveries to the detention center had a detrimental effect on youths’ self-care while in detention.

#### Subtheme 4c: Youth placed in quarantine upon entry at the detention center

Youth expressed the impact quarantine had on their detention center stay. Youth were placed in quarantine upon entry at the detention center. After the youth completed detention center intake, they were required to quarantine in an isolated part of the center to minimize the risk of new youth introducing COVID-19 at the center. The two detention centers participating in the study had slightly different quarantine protocols. One center required youth to quarantine in a group in a separate part of the detention center; the other required youth to be in an individual cell for the length of quarantine. A participant described the feeling of freedom that came upon completion of his quarantine period: "When we first get in here, for everybody, you be in quarantine, and it’s like a whole side, away from the actual cells. So, you have to be in there for 10 days. But after you be in there, after those 10 days, you’re really free. You don’t even need to wear a mask." When discussing changes in detention due to COVID-19, a youth shared: “[Quarantine] was 14, but now it’s 10 days. And you just don’t get to do nothing. You just pretty much sit in your cell half the time. Because they were really low on staff, they’re always with the kids that aren’t in quarantine. When I was on quarantine, I’d get put in my cell about 12 o’clock, lunchtime, and I wouldn’t come out until the next day. So, it’s irritating.” Another youth shared the sentiment of isolation, “But quarantine, it was just like you being stuck in one place all day.” A youth described the effect of quarantine on her mental health: "Even though it’s a detention center, we’re still kids…They do not do a good job of making us feel happy or anything…we sit here, and we watch the same things over and over again…It’s just the same cycle over and over again. And that really puts a strain on your mind, especially if you have mental issues…" Quarantine was standard during the pandemic and was widely implemented in detention centers.

Collectively, the viewpoints of the participants shed light on the attitudes of young people in detention regarding the COVID-19 pandemic and highlight the critical need for reliable and accurate information in addition to attention to balancing the negative impacts of pandemic mitigation strategies on youths’ relationships, self-care, and mental health. Themes and subthemes are summarized in Table 2.

**Table 2:** Themes and subthemes.

**Theme 1: Personal experience influenced perceptions of pandemic severity and risk.**
Subtheme 1a: COVID-19 is a significant issue.
Subtheme 1b: COVID-19 is fictitious or not a serious public health concern.
**Theme 2: Distrust and misconceptions contribute to vaccine hesitancy or refusal.**
Subtheme 2a: Concern about the speed of vaccine development contributed to distrust.
Subtheme 2b: Misconceptions about potential vaccine side effects
**Theme 3: Desired opportunities and parental opinion motivated youth to get the COVID-19 vaccine.**
**Theme 4: Pandemic mitigation strategies negatively impacted detention center experience.**
Subtheme 4a: Limits on visitation and contact while detained during the pandemic.
Subtheme 4b: Limitations on delivery of outside items during the pandemic.
Subtheme 4c: Youth placed in quarantine upon entry at the detention center.

## Discussion

The youth in our study encountered the pandemic in an environment distinct from that of the general population and exhibited insightful knowledge about their pandemic experience. This study addresses a critical gap in our current understanding of this impact of incarceration on public health by exploring the lived experiences and perceptions of youth detained during the pandemic. Although most participants acknowledged the seriousness of the pandemic, they also expressed vaccine mistrust and hesitancy. Some youth believed the pandemic to be fictitious and modeled their behavior accordingly. Further, youth shared the negative impact of pandemic mitigation efforts in detention settings on their mental health and relationships. Together, these findings suggest opportunities to reduce pandemic-associated health disparities among youth in detention.

Our findings align with previous studies that found that although some youth were knowledgeable about the COVID-19 pandemic, others cited inaccurate or non–evidence-based information (e.g., "COVID is man-made," "it is a stronger strain of the flu") [14]. Misinformation expressed by our participants centered on vaccination and the virus’s origin. In contrast to previous work showing that youth in the general population received COVID-19 information mainly from news outlets and scientific, medical, and healthcare sources, the youth in our study primarily received information from family, friends, and social media [15]. Our findings suggest that although social media impacted how detained youths navigated the pandemic, familial experiences and influence significantly contributed to their willingness or unwillingness to get the COVID-19 vaccine. These results are similar those in the incarcerated adult population showing that most of the participants were not comfortable getting the COVID-19 vaccine because of distrust about its development and concerns about long‐term adverse effects [16].

Vaccination is a crucial strategy to curb the pandemic and minimize the risk of severe illness and death [17]. Limited data are available on COVID-19 vaccination rates of youth detention facilities. As of September 2021, only six states had published COVID-19 vaccination numbers of youth in detention, with most reporting that under 50% of their youth population was fully vaccinated [18]. Studies focusing on the U.S. general adolescent population have shown that youth are willing to be vaccinated when they feel vaccination is safe and efficacious [15, 19]. Among a national sample of U.S. youth, 81% were willing to get the vaccine only if trusted experts deemed the vaccination safe. Among the detained youth in our study, a strong mistrust in the swiftness of COVID-19 vaccine development contributed to an unwillingness to get the vaccine. It is not readily apparent that the decreased vaccine acceptance was directly related to their detention status. However, vaccination unwillingness among incarcerated adults has been linked to younger incarcerated individuals (18–29 years old) and those who lived in jails [20]. Studies have highlighted the use of youth-centered materials that directly address vaccine safety, side effects, and efficacy to dispel the fears of youth [15]. Our findings add to this literature by suggesting that vaccine uptake by youth in detention may also be improved by highlighting personal gains such as sports participation or travel for vaccinated youth. Leveraging these vaccine motivators may facilitate increased COVID-19 or future novel vaccination in this population, which in turn can contribute to decreased health inequities.

People living in detention facilities are at higher risk of contracting COVID, taking a devastating toll on an already strained detention system [6]. Although detention facilities needed to alter protocols to reduce the spread of COVID-19, our study suggests that these modifications may have negatively impacted youths’ experience in detention and contributed to secondary negative impacts of the pandemic. The nature and design of detention facilities make it difficult to implement public health measures to mitigate the spread of the virus. Overcrowding, inadequate infection control measures, and lack of testing have fueled massive COVID-19 outbreaks in U.S. detention facilities [5]. Our participants shared that infection control measures such as phone-only visitation, lack of physical touch during in-person visitation, and quarantine contributed to feelings of confinement and isolation. These measures may exacerbate the already profound mental health morbidity among the adolescent justice population [2]. One strategy to address this problem may be a firm commitment to implementing decarceration, which has been shown to reduce COVID-19 risk at the federal level [5]. Although infection control and adequate screening practices are essential risk mitigation strategies, decarceration or alternative sentencing may lower both risks of COVID-19 transmission and the risk for secondary negative impacts such as isolation related to infection control practices [2, 5].

Overall, our findings suggest that youths’ perception of the seriousness of the pandemic impacted their decision to take action to decrease their risk related to the pandemic. Detained youth may not practice preventive measures, including social distancing, mask-wearing, hand hygiene, and vaccination against COVID-19, if they are healthy or if their experience with the virus has not yielded severe morbidity or mortality. Understanding that living in a confined setting increases the risk of contracting and spreading the virus and that unvaccinated individuals are at greater risk for significantly poor health outcomes associated with pandemics may increase motivation to use risk mitigation measures. Youth recognition that preventive measures will reduce their health risks without adverse side effects may increase actions to reduce pandemic-associated risk. Targeted strategies (e.g., social media) could be used to address misinformation about pandemics and vaccination, encourage safe health practices, and affirm youths’ ability to successfully navigate pandemics in or out of detention.

This study has the following limitations. Generalizability is limited as we enrolled youth in a single Midwest city; however, we increased heterogeneity by recruiting from two detention centers as our metropolitan area crosses the state border. Most participants were male, reflecting the detention center populations. The sample size was dependent on detention center capacity at the time of the study. Despite a small sample size, we reached data saturation among our sample overall [12], unique ideas among subgroups may have been missed. Because participants were in short-term detention, their perspectives on the pandemic may have been influenced by their experiences with COVID-19 both inside and outside of the detention setting.

Our findings demonstrate that centering the voices of youth in detention around their pandemic experience identifies opportunities to mitigate health risks, minimize secondary impacts of the pandemic, and reduce pandemic-associated health disparities among this population. Further studies should explore insights of detention facility staff on impacts of the pandemic and associated detention center policies and practices and engage youth in detention and detention staff to formulate protocols, support services, and educational tools to decrease infection, spread, and impact of pandemics among youth in detention.

## Supporting information

S1 File Demographic data of study participants. Demographic survey raw data for study participants, including age, gender, and ethnicity. This dataset provides an overview of the sample population from two juvenile detention centers in the urban Midwest.
(XLSX)

S2 File REDCap demographic survey instrument. REDCap form utilized for gathering demographic details from study participants.
(PDF)
