# Supplementary material for: Pandemic perspectives from detained youth during COVID-19: Bridging the knowledge gap for future safeguards
Source: PLoS One. 2024 Oct 9;19(10):e0309179. doi: 10.1371/journal.pone.0309179 (PMC11463777; doi:10.1371/journal.pone.0309179)
Supplement: S2 File — REDCap form utilized for gathering demographic details from study participants. (PDF) [file pone.0309179.s002.pdf]

Healthcare Needs and Experiences of Detained Adolescents

How old are you? (age in years)

◯ 14 ◯ 15 ◯ 16 ◯ 17

What is your ethnicity?

◯ Hispanic or Latino/Latina/Latinx
◯ Non- Hispanic or non-Latino/Latina/Latinx

What is your race?

◯ American Indian or Alaska Native
◯ Asian
◯ Black or African American
◯ Native Hawaiian or Other Pacific Islander
◯ White
◯ Mixed-Race
◯ Unknown / Unsure
◯ Prefer Not to Answer

Please specify your mixed-race:

____

What is your gender?

◯ Female
◯ Male
◯ Non-binary / Third gender
◯ Prefer to self-describe
◯ Prefer not to say

Please describe your gender:

____

What is the last grade you completed?

____

Do you have a primary care doctor or a designated place you go to for well checks?

◯ Yes ◯ No

Where did you go the last time you needed health care?

◯ Primary care office (Pediatrician, Family Medicine, internal medicine)
◯ Emergency Department
◯ Other

Where did you go the last time you needed health care?

When was the last time you saw a healthcare provider for a regular checkup?

◯ During my current stay at this detention center.
◯ Within the last 12 months NOT including any checkup I have had during my current stay at this detention center.
◯ More than 12 months ago.
◯ I can't remember.

Please click submit below, then hand the tablet to the doctor.
